# Effect of pyrroloquinoline quinone on skin aging in Bmi-1 KO mice and underlying mechanisms

**DOI:** 10.1371/journal.pone.0319770

**Published:** 2025-03-28

**Authors:** Bin Li, Xiao Meng Yang, Xiong Ming Zhou, Yuan Qing Huang

**Affiliations:** 1 Department of Stomatology, Hunan University of Medicine, Huaihua, China; 2 Department of Sonography, Hunan University of Medicine General Hospital, Huaihua China; 3 Department of Oral and Maxillofacial Surgery, The First Affiliated Hospital of Nanchang University, Nanchang, China; NMIMS Deemed to be University - Mumbai Campus: NMIMS, INDIA

## Abstract

To investigate the effect of pyrroloquinoline quinone (PQQ) on skin aging in the Bmi-1 KO mice and its underlying mechanisms, we administered a normal diet to both Wild type mice (WT) and Bmi-1 KO mice, while supplementing the diet of Bmi-1 KO mice with PQQ (PQQ+Bmi-1 KO). Subsequently, we compared the thickness of the skin epidermis, dermis, pilosebaceous unit and collagen ratio using HE staining and Masson’s trichrome. Additionally, immunohistochemical staining, Western blotting and electron microscopy were applied across all three groups. The results revealed that Bmi-1 KO mice exhibited premature aging phenotypes compared to the WT group; however, PQQ administration effectively delayed premature aging in Bmi-1 KO mice. Furthermore, reduced epidermal thickness, dermal thickness, pilosebaceous units count as well as collagen ratio were observed in Bmi-1 KO mice. Moreover, the PCNA positive cell percentage also decreased in Bmi-1 KO mice. Conversely, treatment with PQQ significantly increased epidermal thickness, dermal thickness, pilosebaceous unit count, collagen ratio and PCNA positive cell percentage when compared to Bmi-1 KO mice. In order to further investigate the anti-aging mechanism of PQQ, experiments have revealed that PQQ effectively suppressed the expression of cell cycle proteins p16, p19, and p53 in Bmi-1 KO mice. In addition, autophagy-related experiments demonstrated that compared to the WT group, Bmi-1 KO mice exhibited an increased number of autophagosomes along with decreased expression of Beclin-1 and LC3Ⅱ/LC3Ⅰratio, and increased expression of p62. However, supplementation with PQQ resulted in a reduction in the number of autophagosomes while increasing the expression of Beclin-1 and LC3Ⅱ/LC3Ⅰratio and decreasing the expression of p62. This study provides evidence that downregulation of Bmi-1 promotes skin aging, whereas PQQ delays skin aging in Bmi-1 KO mice by promoting cell proliferation, inhibiting the expression of p16, p19 and p53 and enhancing autophagy levels.

## Introduction

As the outermost organ of the human body, the skin is a reflection of overall health and ageing status. As a vital barrier, it plays a crucial role in protection and immunity. However, skin ageing is inevitably an ongoing concern as it is the most obvious external change and is considered to be the result of the combined influences of both internal and external factors. Research into skin ageing has always been at the forefront [1].

The B cell-specific Moloney MLV insertion site-1 (Bmi-1), a crucial member of the Polycomb family, plays a pivotal role in regulating cell proliferation and differentiation, as well as maintaining self-renewal and multidirectional differentiation of normal stem cells [2]. Mechanistically, studies have demonstrated that Bmi-1 gene regulation in stem cells is associated with its suppression of p16^INK4a^ and p14^ARF^ expression, which are closely involved in cell cycle regulation [3]. p19ARF proteins (p14^ARF^ homologs) can stabilize p53 by antagonizing the ubiquitin protein ligase murine double minute 2 (MDM2) and p53-dependent transcription, resulting in cell cycle arrest in G1 and G2/M phases. p16^INK4a^ and p19^ARF^ have been confirmed as important targets of Bmi-1 action. Bmi-1 promotes cell proliferation by inhibiting the p16/RB and p19^ARF^/MDM2/p53 pathways [4,5]. Furthermore, our previous study revealed that deletion of the Bmi-1 gene led to tibial senescence in mice [6].

However, whether knockout of the Bmi-1 gene induces skin aging in mice and its underlying mechanisms remain to be further investigated.

During the aging process, in order to maintain physiological stability, the skin undergoes continuous renewal of the epithelial compartment and homeostasis of long-lived cell types in response to external stresses to maintain physiological stability [7]. Recent studies have shown that the maintenance of skin homeostasis is closely linked to autophagy and that the onset of skin ageing is associated with changes in autophagy activity [8,9]. Similar observations have been made in other tissues such as brain cells undergoing aging, where there is downregulation of autophagy-related gene expression [10]. Impaired autophagy activity can lead to accumulation of senescence-associated modified proteins and damaged organelles in cells, subsequently inducing a senescent phenotype [11,12].

Pyrroloquinoline quinone (PQQ), a bacterially synthesized compound, is widely distributed in plants, bacteria, animals, food, and various biological fluids. It plays a crucial role in growth and development [13,14]. Dietary intake adequately meets the physiological requirements PQQ to of the human body. Scientific evidence has demonstrated the neuroprotective and cardioprotective effects of pyrroloquinoline quinone [15,16]. PQQ effectively mitigates the accumulation of reactive oxygen species and has anti-aging properties [17,18]. Therefore, the aim of this article is to investigate the potential anti-aging effects of PQQ and its underlying mechanisms in Bmi-1 KO mice.

## Materials and methods

Homozygous Bmi-1 KO mice and wild-type littermates were obtained and genotyped as previously described [6]. The mice were housed in a temperature-controlled facility with a 12-hour light/dark cycle, according to the guidelines of the Institute for Laboratory Animal Research at Nanjing Medical University. The study was approved by the Committee on the Ethics of Animal Experiments at Nanjing Medical University (Permit Number: BK2006576). Purified PQQ was generously provided by Professor Chuan jun Wen from Nanjing Normal University. The experiment consisted of three groups: Wild-type group (WT): six weaning littermate wild-type mice aged three weeks were fed a normal diet for four weeks. Bmi-1 KO group: six weaning littermate Bmi-1 KO mice aged three weeks were fed a normal diet for four weeks. PQQ-supplemented diet group: six weaning littermate Bmi-1 KO mice aged three weeks were fed a PQQ-supplemented diet (4 mg PQQ/kg in normal diet) for four weeks.

### Analysis of phenotype and body weight

The effect of PQQ was assessed by examining the phenotypic changes and body weight changes in three groups.

### H&E and Masson’s staining

Mice in each group were euthanized by cervical dislocation, and the dorsal skin was collected and fixed in 4% paraformaldehyde. Subsequently, the skin samples were embedded in paraffin for section preparation. Hematoxylin and eosin (H&E) staining, following established protocols [19], was performed to quantify the thickness of the epidermis, dermis, and sebaceous glands. Masson’s staining was employed to evaluate collagen quality and distribution [20]. Finally, stained tissue sections were examined under an optical microscope (BX51, Olympus, Japan).

### Immunohistochemical staining

Skin samples were fixed in 4% paraformaldehyde for 24 hours, dehydrated and embedded in paraffin. After deparaffinization and rehydration of the sections, they were treated with 3% H_2_O_2_ for 10 minutes. The sections were then blocked with 5% normal serum for 20 minutes before being incubated separately overnight with a primary antibody against PCNA (1:400, Abcam, UK). Following rinsing, the sections were incubated with a secondary antibody (1:100, Abcam, UK) for 20 minutes and exposed to DAB solution (Boshide, Wuhan ，China) at room temperature for 5–8 minutes. Finally, the sections were counterstained with H&E staining for one minute and then imaged under a microscope. The percentage of PCNA-positive cells was evaluated as the ratio of positive nuclei to total nuclei per field (at a magnification of × 400) in both the basal epidermal layer and dermal fibroblasts by assessing at least five randomly selected fields per mouse.

### Western blot analysis

Dorsal skin was used for protein extraction, followed by quantification. Subsequently, the proteins were separated using 10%-12% SDS-PAGE gel electrophoresis. Afterwards, the PVDF membrane was incubated overnight at 4 °C with primary antibodies: Beclin1 (1:800, Abcam, UK), LC3 (1:1000, Abcam, UK), p62 (1:800, Abcam, UK), p16 (1:800, Santa Cruz Biotechnology, USA), p19 (1:800, Abcam, UK), and p53 (1:1000, Abcam, UK). Following three rinses with Tris-buffered saline solution, the PVDF membrane was incubated with a secondary antibody (Sigma, St. Louis, MO, USA) for one hour. Finally, the bands were visualized using enhanced chemiluminescence (ECL, Amersham) and analyzed using Image Quant-LAS-4000 mini.

### Electron microscope

As reported [21], the expression of autophagosomes was checked by electron microscopy. After the mice were anesthetized with ether, the dorsal skin tissues were cut into l mm ×  2 mm strips and immediately immersed in 3% glutaraldehyde for 4 h, then rinsed with PBS for 10 min and treated with 1% osmic acid for 2 h. The skin samples were dehydrated in gradient ethanol, replaced with epoxy resin Epon812 embedding agent mixture (1:1), and infiltrated overnight at 37°C. After polymerization of the epoxy resin, the skin slices were cut at 40–60nm, and stained with uranyl acetate and lead citrate. After drying, images were examined by Zeiss 900 transmission electron microscope equipped with Olympus Morada G2 digital cameras at ×  30,000 magnification.

### Statistic analysis

The results of all analyses were reported as the mean ±  SEM (standard error of the mean). Comparisons between two groups were assessed using Student’s t-test. A p-value less than 0.05 was considered statistically significant. Statistical analyses were analyzed by SPSS software (IBM, SPSS Statistics, US. version 23).

## Results

**Fig 1:**
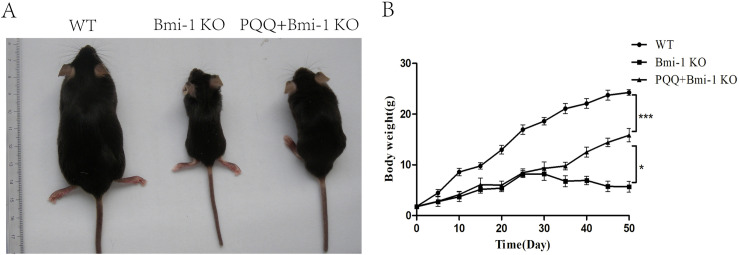
Effect of PQQ on phenotype and body weight in Bmi-1 KO mice. (A) Effect of PQQ-supplemented diet on body size of Bmi-1 KO mice. (B) Effect of PQQ-supplemented diet on body weight of Bmi-1 KO mice. (Body weight changes after feeding. n = 6 WT mice, n = 6 Bmi-1 KO mice and n = 6 PQQ+Bmi-1 KO mice). Values are group mean ±  SEM, t-Test: * , ** and *** indicate *P* < 0.05, *P* < 0.01 and *P* < 0.001, respectively.

To investigate the potential of PQQ in delaying premature aging phenotype in Bmi-1 KO mice, we evaluated the phenotypic changes (Fig 1A) and body weight alterations (Fig 1B) across three experimental groups. The results revealed that compared to wild-type (WT) mice, Bmi-1 KO mice exhibited a decrease in total body size and a loss of body weight (*P* < 0.001). However, administration of PQQ resulted in a restoration of body size and an increase in body weight in Bmi-1 KO mice (*P* < 0.05).

### Effect of PQQ on skin aging in Bmi-1 KO mice

**Fig 2:**
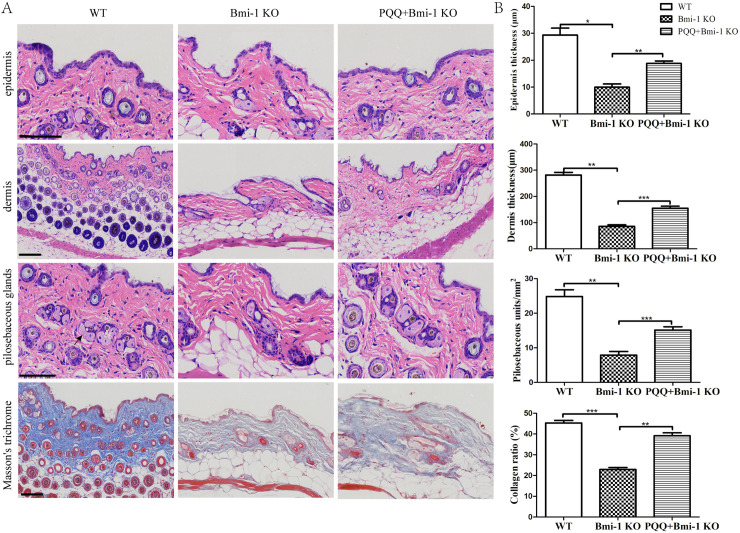
Effect of PQQ on skin aging in Bmi-1 KO mice. (A) Representative H&E and Masson’s trichrome-stained skin sections of WT mice, Bmi-1 KO mice and PQQ+Bmi-1 KO mice. (HE staining was used to detect epithelial thickness, dermal thickness, and number of sebaceous glands in mice (black arrows represent sebaceous glands in the figure), and Masson’s trichrome-stained to detect collagen bundles). (B) Bar graphs show semiquantitative evaluation of epidermal and dermal thickness, pilosebaceous unit and ratio of collagen bundles (n = 6 WT mice, n = 6 Bmi-1 KO mice and n = 6 PQQ+Bmi-1 KO mice). Scale bar: 100µm. Values are group mean±SEM, t-Test: * , ** and *** indicate *P* < 0.05, *P* < 0.01 and *P* < 0.001, respectively.

The detection of skin ageing parameters in gene knockout mice was reported as previously described [22]. H&E-stained skin sections (Fig 2A and B) revealed a significant reduction in both epidermal and dermal thickness in Bmi-1 KO mice compared to the wild-type (WT) group (*P* < 0.05, *P* < 0.01 respectively). Additionally, a notable decrease in pilosebaceous unit was observed in Bmi-1 KO mice (*P* < 0.01). Masson’s trichrome staining demonstrated that collagen bundles became less dense and significantly decreased in Bmi-1 KO mice (*P* < 0.001). In contrast, treatment with PQQ resulted in an increase of epidermal thickness (*P* < 0.01) compared to the Bmi-1 KO group, as well as an increase in dermal thickness (*P* < 0.001) and the number of pilosebaceous unit (*P* < 0.001). Furthermore, Masson’s staining showed that supplementation with PQQ led to an increased ratio of collagen bundles compared to those observed in Bmi-1 KO mice (*P* < 0.01), in addition, the density of the collagen bundles was increased in PQQ-fed Bmi-1 mice.

### Effect of PQQ on PCNA in Bmi-1 KO mice

**Fig 3:**
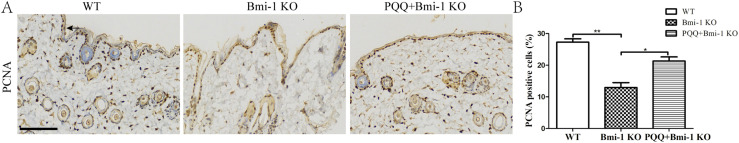
Effect of PQQ on PCNA in Bmi-1 KO mice. (A)Representative images of PCNA immunostaining of skin from WT mice, Bmi-1 KO mice and PQQ+Bmi-1 KO mice. (B) Bar graphs show semiquantitative evaluation of PCNA positive cells in WT mice, Bmi-1 KO mice and PQQ+Bmi-1 KO mice (Brown-stained are PCNA cells, The percentage of PCNA-positive cells was evaluated as the ratio of positive nuclei to total nuclei per field. n = 6 WT mice, n = 6 Bmi-1 KO mice and n = 6 PQQ+Bmi-1 KO mice). Scale bar: 100µm. Values are group mean±SEM. t-Test: * , ** and *** indicate *P* < 0.05, *P* < 0.01 and *P* < 0.001, respectively.

In order to elucidate the relationship between skin aging due to Bmi-1 gene deletion and impaired skin cell proliferation capacity, we performed an in vivo evaluation of cell proliferation using PCNA immunohistochemistry. The results of PCNA immunostaining (Fig 3A and B) revealed a significant decrease in Bmi-1 KO mice compared to WT mice (*P* < 0.01). However, supplementation with PQQ resulted in an increase in PCNA-positive cells compared to Bmi-1 KO mice (*P* < 0.05).

### Effect of PQQ on cell cycle proteins of in Bmi-1 KO mice

**Fig 4:**
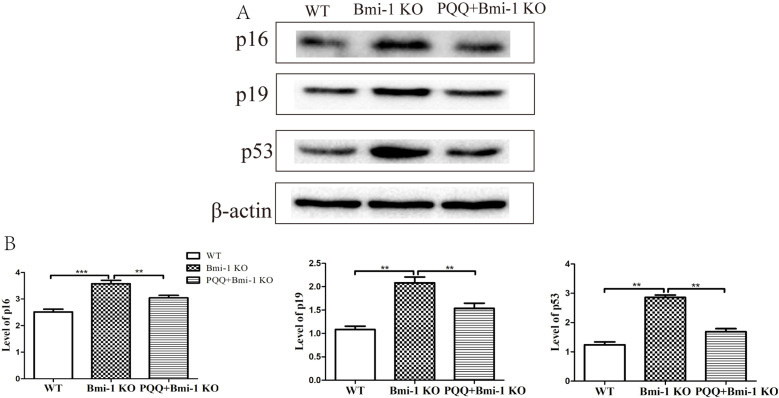
Effect of PQQ on skin cell cycle proteins in Bmi-1 KO mice. Representative Western blots of cell extracts for the expression of p16, p19 and p53. β-actin was used as loading control for Western blots in WT mice, Bmi-1 KO mice and PQQ +  Bmi-1 KO mice respectively; p16, p19 and p53 protein levels relative to β-actin protein levels were assessed by densitometric analysis. (n = 6 WT mice, n = 6 Bmi-1 KO mice and n = 6 PQQ+Bmi-1 KO mice).Values are group mean ±  SEM. t-Test: * , ** and *** indicate *P* < 0.05, *P* < 0.01 and *P* < 0.001, respectively.

The regulation of proliferation involving cell cycle proteins, such as p16, p19 and p53, is widely recognized. To investigate the potential regulatory role of PQQ on cyclins, we conducted western blot experiments to examine these proteins. The results revealed a significant increase in the expression levels of p16 (*P* < 0.001), p19 (*P* < 0.01), and p53 (*P* < 0.01) in Bmi-1 KO mice compared to WT mice. However, supplementation with a PQQ-enriched diet in Bmi-1 KO mice led to a significant decrease in the expression levels of p16 (*P* < 0.01), p19 (*P* < 0.01), and p53 (*P* < 0.01). These findings are depicted in Fig 4A and B.

### Effect of PQQ on autophagy in Bmi-1 KO mice

**Fig 5:**
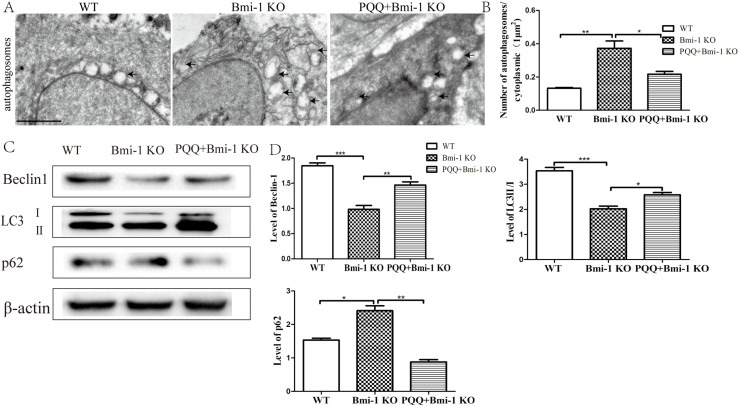
Effect of PQQ on autophagy in Bmi-1 KO mice. (A) Representative images of the number of autophagosomes from WT mice, Bmi-1 KO mice and PQQ+Bmi-1 KO mice. (B) Under TEM, the number of autophagosomes per 1μm^2^ cytoplasmic area were 0.13 ± 0.004, 0.37 ± 0.04, 0.22 ± 0.02 in WT mice, Bmi-1 KO mice and PQQ +  Bmi-1 KO mice respectively; (black arrows represent autophagosomes. n = 6 WT mice, n = 6 Bmi-1 KO mice and n = 6 PQQ+Bmi-1 KO mice). Scale bar: 1000µm. Values are group mean ±  SEM. t-Test: * , ** and *** indicate *P* < 0.05, *P* < 0.01 and *P* < 0.001, respectively. (C) Representative Western blot for expression of Beclin-1, LC3Ⅱ/LC3Ⅰratio and p62. β-actin was used as loading control for western blot in WT mice, Bmi-1 KO mice and PQQ +  Bmi-1 KO mice respectively; (D) Beclin-1 and LC3Ⅱ/LC3Ⅰratio and p62 proteins levels relative to β-actin protein levels were assessed by densitometric analysis. Values are group mean ±  SEM. t-Test: * , ** and *** indicate *P* < 0.05, *P* < 0.01 and *P* < 0.001, respectively.

To further elucidate the underlying mechanisms of skin aging in Bmi-1 KO mice and the anti-aging effects of PQQ, we investigated the expression levels of autophagosomes and autophagy-related proteins. Electron microscopy analysis (Fig 5A and B) revealed a significant increase in the number of autophagosomes in Bmi-1 KO mice compared to WT mice (*P* < 0.01), whereas treatment with PQQ resulted in a reduction in the number of autophagosomes compared to Bmi-1 KO mice (*P* < 0.05). Western blot analysis (Fig 5C and D) demonstrated a down-regulation of Beclin-1 expression (*P* < 0.001) and LC3Ⅱ/LC3Ⅰratio (*P* < 0.001), and an up-regulation of p62 expression (*P* < 0.05) in Bmi-1 KO mice when compared to WT mice. Conversely, treatment with PQQ led to an upregulation of Beclin-1 expression (*P* < 0.01) and LC3Ⅱ/LC3Ⅰratio (*P* < 0.05), together with a decrease in p62 expression levels (*P* < 0.01). These findings suggest that knockout of the Bmi-1 gene can modulate autophagy levels, while PQQ may counteract skin aging by enhancing autophagic activity.

## Discussion

In this study, we have confirmed that deletion of the Bmi-1 gene accelerates skin aging through decreased proliferation. In addition, supplementation of PQQ effectively reverses the skin aging caused by Bmi-1 deficiency. Our findings suggest a crucial role for PQQ in delaying aging by promoting cell proliferation, inhibiting the expression of p16, p19 and p53 and upregulating autophagy levels in the skin of Bmi-1 knockout mice.

The Bmi-1 gene, a member of the Polycomb group gene family, has been extensively implicated in the regulation of cell cycle progression, aging processes, stem cell self-renewal, and other essential biological mechanisms [23]. Numerous studies have consistently demonstrated that decreased expression of Bmi-1 in brain tissue is associated with degenerative brain diseases such as Alzheimer’s disease (AD) and Parkinson’s disease (PD) [24,25]. Moreover, dysregulated expression of Bmi-1 can lead to mitochondrial dysfunction, thereby promoting cellular aging [26]. Our previous investigations have provided evidence that deletion of the Bmi-1 gene induces senescence in mice tibiae [6]. Similarly, Zhao J et al. found that Bmi-1 epigenetically orchestrates osteogenic and adipogenic differentiation of bone marrow mesenchymal stem cells to delay bone aging [27]. Therefore, Bmi-1 has emerged as a crucial biomarker for age-related diseases; while its precise role in skin aging remains partially elucidated. In this study, we found that Bmi-1 KO mice exhibited a decrease of overall body size and a loss of body weight, when compared to wild-type (WT) mice. It suggested that deletion of the Bmi-1 gene leaded to impaired growth and development in mice. To gain further insights into its involvement in skin aging processes, we examined the onset and severity of age-related skin changes in Bmi-1-deficient mice using histological analysis with H&E staining which revealed a reduction in epidermal thickness and pilosebaceous unit count. Additionally, Masson’s trichrome staining revealed a decrease in collagen bundles in Bmi-1 knock-out mice compared to WT mice. Considering that age-related epidermal thinning and dermal atrophy are attributed to reduced turnover rates of keratinocytes and dermal fibroblasts, we investigated the effect of Bmi-1 gene deficiency on cutaneous cell proliferation in vivo using immunohistochemistry. PCNA as a marker of cellular proliferation, in our study, immunostaining for PCNA demonstrated a reduction in Bmi-1 KO mice compared to WT mice. Furthermore, Western blot analysis was conducted to assess the expression levels of p16, p19 and p53, which regulate cell proliferation and reflect cellular aging. The results indicated that deletion of the Bmi-1 gene promoted the expression of p16, p19 and p53. Overall, our study suggests that deletion of the Bmi-1 gene accelerates skin aging. On this basis, we have been trying to find an effective anti-ageing agent, and PQQ is an important breakthrough point in our research.

Pyrroloquinoline quinone, as a vitamin-like cofactor that the human body can obtain through food, is important for health and disease prevention, and it has a good biosafety profile. Currently, oral administration is one of the more common methods of PQQ administration, both in animal and human trials. As for the selection of the concentration of PQQ, it is related with the species of animals, animal models, routes of administration, and the purpose of the study. The concentration of pyrroloquinoline quinoline in our previous and Gen’s studies [6,28], we found that Bmi-1 knockout mice showed significant changes in growth status, body weight changes, activity responses, and some aging-related indices at a concentration of 4 mg/kg. In addition, Steinberg, F.M found that mice could obtain growth and development at the concentration of 0.3–5mg/kg when studying the effect of PQQ on BALB/c mice. In recent years, a large body of evidence has shown that PQQ is a potent antioxidant that modulates the number and function of mitochondria in aged rats and ameliorates oxidative stress and lipid peroxidation in the brain [13,29]. Of particular note, in human-related studies, PQQ supplementation significantly improved the skin condition of female subjects with dry skin [30]. PQQ was found to be protective against uva radiation-induced senescence of human dermal fibroblasts in an in vitro study [31]. The potential of PQQ in delaying aging is highly esteemed, as it can ameliorate D-galactose-induced cognitive impairment by attenuating glutamate neurotoxicity through the GSK-3β/Akt signaling pathway in mice [28]. PQQ exhibits inhibitory effects on the p16^INK4a^/Rb and p19^AFR^/p53 pathways implicated in cell cycle regulation [32]. And our study revealed similar findings as we observed that PQQ administration led to the restoration of body size and an increase in body weight among Bmi-1 KO mice. Additionally, it effectively enhanced epidermal thickness, dermal thickness, pilosebaceous unit count, collagen ratio, and PCNA positive cell percentage compared to Bmi-1 KO mice. PQQ demonstrated the ability to suppress the expression of cell cycle proteins p16, p19, and p53 in Bmi-1 KO mice. These findings suggest that PQQ can modulate the p16 and p53 pathways to exert anti-aging effects.

During the aging process, cells experience a significant accumulation of damaged molecules and organelles, which is a common characteristic observed in senescent cells and leads to reduced cellular viability. Autophagy, also known as “self-phagocytosis,” allows cells to utilize lysosomes to degrade their own damaged organelles and macromolecular substances, regulating and monitoring the protein and organelle quality within the cell. The process of autophagy maintains intracellular homeostasis, thereby exerting a cell-autonomous mechanism to suppress aging [33]. Moreover, autophagy contributes to diverse cellular functions that safeguard neighboring cells, including facilitating the differentiation of epithelial cells and enhancing their defense against external noxae [34]. Furthermore, autophagy is thought to play a crucial role in maintaining nutritional sensitivity and genomic stability—both recognized hallmarks of aging [35]. Similarly, experimental evidence suggests that autophagy plays a pivotal role in maintaining skin homeostasis, and the process of cutaneous aging is associated with and partly caused by autophagy dysfunctions [36]. For instance, in a fruit fly skin aging model, it was found that with increasing age, the epidermis of the fruit fly undergoes a series of morphological deteriorations, including damage to the cell membrane and nucleus. These changes were slowed down in long-lived mutants and accelerated in short-lived mutants. An increase in autophagy markers associated with epidermal cell ageing was detected. The Atg7 mutant was a short-lived mutant and its epidermal layer retained youthful characteristics, indicating that autophagy plays an important role in epidermal aging and has a protective effect [37]. Considering the pivotal role of autophagy in skin aging, we investigated the expression of autophagy-related proteins and autophagosomes in Bmi-1 knockout mice. Compared to the WT group, Bmi-1 KO mice exhibited an increased abundance of autophagosomes accompanied by decreased Beclin-1 expression and a reduced LC3Ⅱ/LC3Ⅰ ratio, as well as elevated p62 expression. These findings suggest a decrease in autophagic activity in Bmi-1 knockout mice. To counteract this change, we treated the mice with PQQ and observed a decrease in the number of autophagosomes, while simultaneously increasing Beclin-1 levels and LC3Ⅱ/LC3Ⅰ ratio, and decreasing p62 expression. These findings imply that PQQ may attenuate skin aging in Bmi-1 KO mice by promoting autophagic activity.

In conclusion, our data provide evidence supporting the involvement of the Bmi-1 gene in skin aging through its regulation of age-related proteins and cell proliferation. Moreover, supplementation with PQQ demonstrates potential for mitigating these skin alterations by activating autophagy. But there are shortcomings in our research. The study is centered on Bmi-1 knockout mice, which may not fully capture the complexity of skin aging across different genetic backgrounds or in humans. This limits the applicability of the findings to other models or species. Regarding the mechanisms related to skin aging, oxidative stress damage is a very important point, excessive accumulation of intracellular oxidative stress levels is closely related to aging-related diseases such as cancer, skin aging, and so on. The accumulation of reactive oxygen species can induce senescence in the organism. So further investigations are warranted to elucidate the underlying mechanisms by which PQQ modulates autophagy, including oxidative stress and mitochondrial function. Only a comprehensive understanding of this complex mechanism can enhance the anti-aging efficacy of PQQ on the skin.

## Supporting information

S1 Data S1_raw_images.pdf. Original image of protein Western blots experiment. (PDF)
