# Supplementary material for: Effect of pyrroloquinoline quinone on skin aging in Bmi-1 KO mice and underlying mechanisms
Source: PLoS One. 2025 Mar 28;20(3):e0319770. doi: 10.1371/journal.pone.0319770 (PMC11952206; doi:10.1371/journal.pone.0319770)

Below are the uncropped gels images from Figure 4 (p16, p19, p53 proteins) and Figure 5 (Beclin1, LC3, p62 proteins), respectively. Each protein includes three sets of reproducible data, and the images of the proteins selected in the article are marked with red boxes, and the unselected ones are marked with X. The molecular weights are also indicated, and each image is labelled with a Marker protein on the left side.

Uncropped gels for Western Blot in fig4

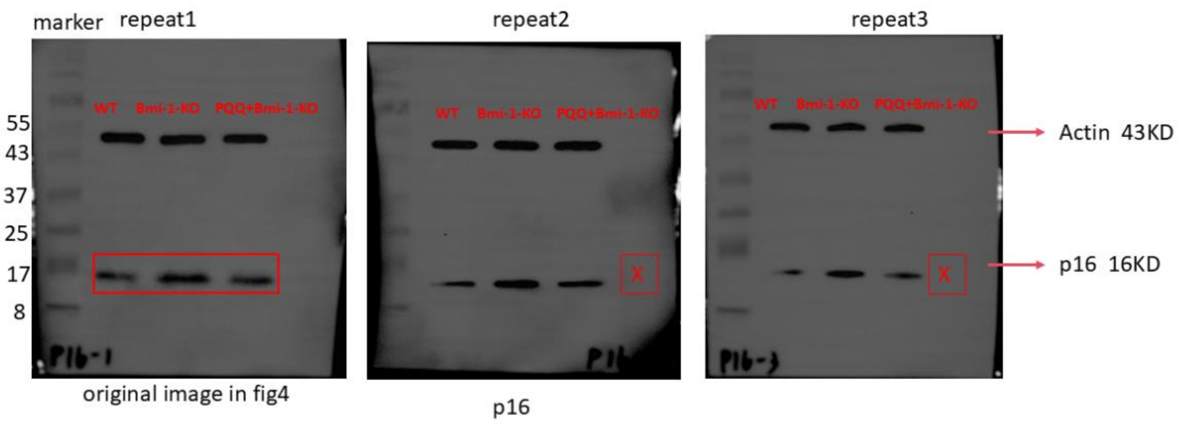

Uncropped gels for Western Blot in fig4

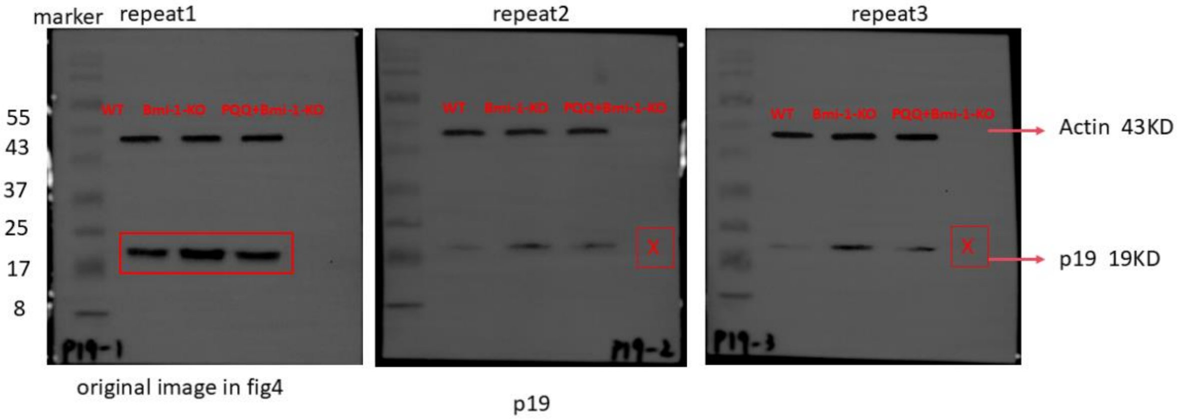

p19

Uncropped gels for Western Blot in fig4

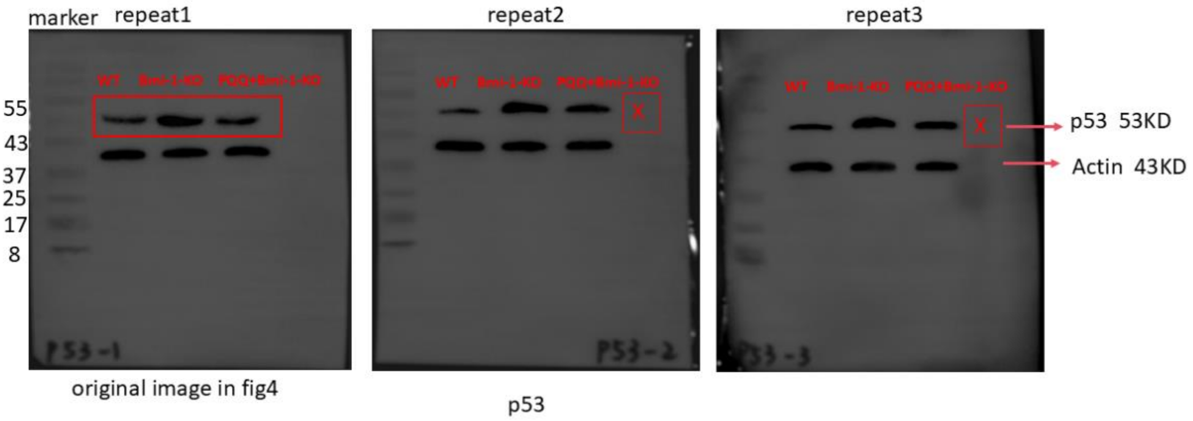

p53

Uncropped gels for Western Blot in fig5

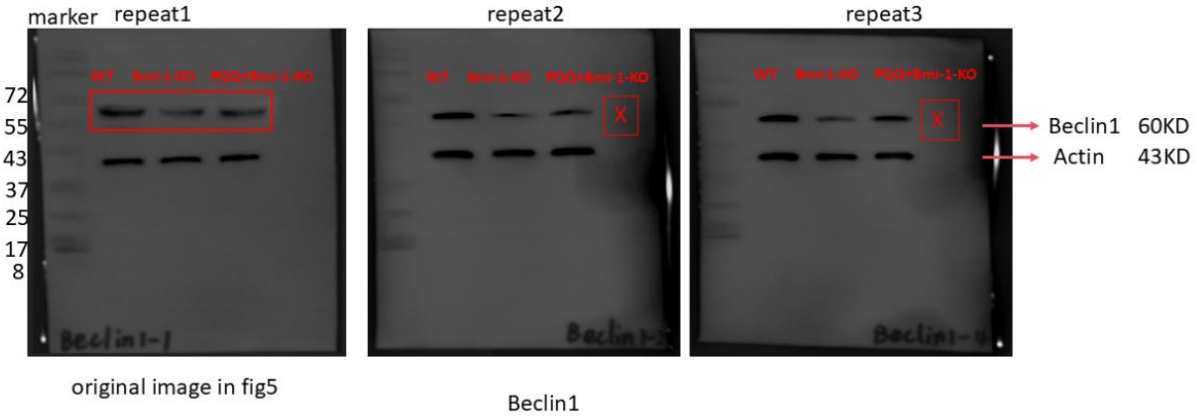

Beclin1

Uncropped gels for Western Blot in fig5

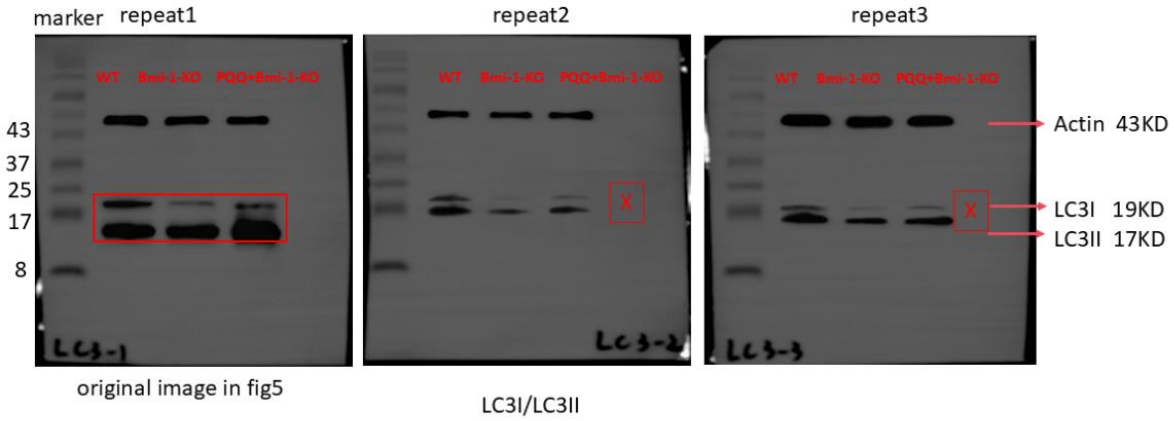

LC3I/LC3II

Uncropped gels for Western Blot in fig5

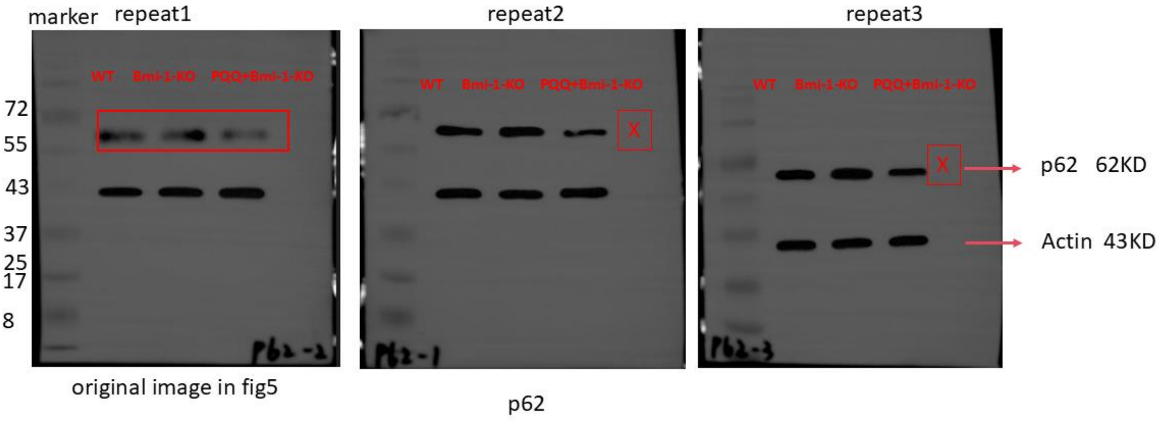

Supplement: S1 Data — (PDF) [file pone.0319770.s001.pdf]
